# Mentalizing in Adolescents with Borderline Personality Disorder

**DOI:** 10.3390/brainsci13101473

**Published:** 2023-10-18

**Authors:** Magdalena Uzar, Monika Dmitrzak-Węglarz, Agnieszka Słopień

**Affiliations:** 1 Department of Child and Adolescent Psychiatry, Karol Jonscher Clinical Hospital, Poznan University of Medical Sciences, Szpitalna 27/33 St., 60-572 Poznan, Poland; agaslopien@ump.edu.pl; 2 Department of Psychiatric Genetics, Medical Biology Center, Poznan University of Medical Sciences, Rokietnicka St. 8, 60-806 Poznan, Poland; mweglarz@ump.edu.pl

**Keywords:** mentalizing, borderline personality disorder, adolescents

## Abstract

Mentalizing, recognized as the capacity to understand behaviors in the context of our own mental states and those of other people, is being researched more and more commonly in regard to various mental disorders. The research on mentalization focuses on, among other things, borderline personality disorder, which is at present perceived as an emerging problem in the population of adolescents. In order to summarize the currently accessible knowledge of mentalizing in adolescents with borderline personality disorder, we thoroughly analyzed relevant publications. Based on the available literature, it can be concluded that the mentalizing ability of adolescents with borderline personality disorder can be impaired. The evidence demonstrates that they are prone to hypermentalizing, defined as an overattribution of mental states to other people. However, this tendency has not been proven to be specific to teenagers with this disorder. Moreover, the existing data suggest that young people with borderline personality exhibit a reduced capacity to mentalize their own inner states.

## 1. Introduction

Borderline personality disorder (BPD) is a serious mental disorder defined as a constant pattern of behavior and emotional experience characterized by instability in interpersonal relationships, variability in self-perception and emotional reactions, and significant impulsiveness [1,2]. There is a growing number of studies that support the validity of diagnosing borderline personality disorder among adolescents [3]. Its etiology, however, is not yet fully understood [2,4]. In research regarding the development of BPD, the occurrence of mentalizing disorder has been investigated [5]. Studies on mentalization and its relationship with borderline personality disorder are based on the concept that BPD at its core may constitute a higher-order mental processing impairment [6,7].

In order to summarize the currently accessible knowledge of mentalizing in adolescents with borderline personality disorder and to provide a multi-faceted insight into this issue, which we believe would be valuable, we thoroughly analyzed relevant publications with the aim of addressing this problem comprehensively, relating to the results of various areas of neuroscience.

We refer to the neurobiological and psychosocial determinants of the ability to mentalize, as well as etiological factors contributing to borderline personality disorder. Furthermore, we describe the development of mentalization and numerous factors that determine it, which include mechanisms that potentially disturb the development of this skill, as well as difficulties specific to adolescents. We also examine characteristics of the clinical picture of borderline personality disorder during adolescence. Our work examines problems related to the evaluation of mentalization in people with this disorder, together with potential factors that may distort the assessment outcomes. Finally, we characterize impairments in the ability to mentalize in adolescents with BPD. We would like to emphasize that our article is not intended to be devoted to treatment/therapy. Hence, there is only a brief reference to this issue.

## 2. Mentalizing

Mentalizing or mentalization has been defined as the higher-order cognitive ability to understand one’s own and other people’s behavior in relation to internal states such as thoughts, feelings, desires, wishes, attitudes, and goals [5,8]. Mentalization is a form of social cognition. It is an imaginative mental activity that allows us to infer mental states [9]. This ability can be viewed from several dimensions: automatic–controlled, cognitive–affective, oriented to internal or external characteristics, as well as oriented to self or others [10]. Currently, mentalizing is regarded as a transdiagnostic and transtheoretical construct, and its impairments are associated with most mental disorders [8]. Mentalizing is considered to be a term that overlaps in meaning with others such as the Theory of Mind, mind reading, empathy, metacognition, mindfulness, perspective taking, and a reflective function [11,12,13].

The development of the capability to mentalize begins very early. In the first months of life, children already exhibit shared attention and common intentionality [8,14,15]. Around 6–9 months of age, or perhaps even earlier, infants begin to understand that behaviors are rational and are intended to achieve specific aims [11]. In this way, children reach a teleological stance that enables them to use the observed reality to comprehend actions’ objectives [11,16]. In the middle of the second year of life, a child begins to show a basic ability to mentalize, by automatic and intuitive understanding that rational, goal-oriented behaviors result from mental states. At 18 months, infants can get involved in pretend play, which, according to some authors, indicates they are able to mentalize [17]. At the age of 3, children acquire the capability of collective intentionality, i.e., they can function in a group, complying with common rules, norms, and conventions [8,14]. Before the age of 4, children learn how to lie, and turning 4, most of them are able to pass the test of false beliefs [11,18]. After age 6, during the school years, children start to understand that people have beliefs about other people and that these beliefs do not necessarily have to be true (second-order false belief competence) [11,19]. In the following years, children improve their ability to lie and deceive others, as well as such skills as irony, sarcasm, and a sense of humor [11,20]. The capacity to mentalize continues to develop during adolescence, coupled with significant structural and functional maturation of the “social brain”, a brain network involved in socio-cognitive processes [21,22]. As a result, it has been recognized that during childhood and adolescence, non-adaptive cognitive strategies such as hypomentalization (insufficient inference about the mental states of others) and hypermentalization (excessive and improper inference) may reflect normative processes of social–emotional learning and adaptation to new, complicated situations in interpersonal relations taking place at that time [22].

The results of research in the field of neuroscience confirm that the ability to mentalize is multidimensional, and within it, the aforementioned four dimensions or polarities can be distinguished, each dependent on a relatively distinct neural circuit [23]. One of these dimensions refers to automatic vs. controlled mentalizing. Automatic or implicit mentalizing is fast and reflexive. It is conditioned by phylogenetically older neural circuits that rely on sensory information [23]. Controlled or explicit mentalizing is conscious, verbal, and reflective. It is based on phylogenetically newer brain circuits connected with linguistic/symbolic processing [23]. The rise in stress or arousal level causes a shift from controlled to automatic mentalizing [8]. On the other hand, available data also indicate that an increased level of emotional arousal can trigger the inhibition of the neural system associated with the ability to mentalize [12,24]. The second dimension of mentalization relates to internal or external characteristics. Externally oriented mentalizing is based on a lateral frontotemporoparietal network and is connected with less reflective processes. The medial frontoparietal network, responsible for more active and controlled reflective processes, underlies internally oriented mentalizing [8,24]. The third dimension regards mentalizing with the self and with others. It is now recognized that common neural circuits are responsible for this dimension of mentalization and can be divided into basic and more advanced, namely a shared representation system (SR), which is based on shared representations of mental states of other people, and a mental state attribution system (MSA), which relies more on symbolic and abstract processing [8,25]. The SR system recruits the inferior frontal gyrus, inferior parietal lobule, anterior insula, and anterior cingulate cortex. MSA is based on a cortical midline system consisting of the ventromedial prefrontal cortex (VMPFC), dorsomedial prefrontal cortex, temporoparietal junction, and medial temporal pole [8,24]. SR and MSA inhibit each other. MSA influences the SR through the top-down regulatory mechanism [8,26].

The development of the ability to mentalize results from both brain development and the individual’s experience, which interact and affect each other [11,22,23]. Mentalization grows on the basis of interactions with other people, and because of that, it is constantly influenced by other people’s mentalizing abilities. Therefore, mentalization is relationship- and context-dependent [8,23].

Previously, the development of mentalizing was considered mainly in the context of the relationship between the child and the primary caregiver [5,12]. It was postulated that secure attachment is a foundation on which the robust mentalizing capacity develops [27]. The role of the primary caregiver’s ability to correctly reflect the child’s mental state was also emphasized [13]. Researchers indicate that the ability to mentalize displayed by a caregiver is a basis for the development of secure attachment in children, as well as children’s capacity to mentalize, which enables proper regulation of emotions and interpersonal functioning [8]. The caregiver’s affective display must be contingent and marked [8,9]. Marked communication refers to caregivers’ capacity to show their child that, on the one hand, they comprehend his or her inner state and that, on the other hand, what they do or say solely concerns the child. They modify their reaction by, for example, exaggerating or slowing down, thanks to which the child acquires the ability to distinguish between his or her own inner state and the caregivers’ expression [11]. The impact of the quality of the relationship between children and the primary caregivers on the development of the mentalizing ability was supported by research indicating that attachment formation disorders, childhood adversity, and traumatic experiences can negatively affect mentalization [6,28,29,30].

Currently, the influence of other factors on the development of mentalizing is also emphasized. Luyten et al. postulate a social–evolutionary communicative model of mentalization development. According to this model, the ability of parents to understand their children’s behavior as determined by their internal states is motivated and strongly related to a broader set of factors influencing a child’s development, such as family, neighborhood, local and national community, and a general socio-cultural context which the child grows up in [8]. The authors of this model underline that the above-mentioned factors affect the evolutionarily conditioned ability to identify knowledge provided by other people as necessary for an individual and generalizable, i.e., used in broader contexts, which enables social learning and salutogenesis, as well as determines secure attachment and proper mentalization [8].

Górska and Marszał point out that mentalizing is connected with emotional arousal resulting from a real or imagined relationship with a person we mentalize with and consequent activation of the attachment system [31]. There is an inseparable connection between mentalization focused on others and that related to one’s own mind. Understanding other people’s mental states allows us to transform and regulate our own emotions [31]. When faced with adversities, secure attachment strategies and mentalizing skills enable children to recalibrate their thoughts and feelings and adapt to a situation by themselves or by interacting with others through co-regulation, which strengthens resilience [8].

Furthermore, it seems important to pinpoint the possible impact of traumatic experiences on the ability to mentalize. The meta-analysis by Sloover et al. based on six studies including 172 adult patients with trauma- and stressor-related disorders shows that mentalizing was significantly and moderately impaired in the patient group in comparison to the control group [32]. Moreover, research on the youth population also demonstrates the occurrence of mentalization disorders in a form of hypomentalizing in teenagers who have experienced childhood trauma [33]. On the other hand, there are also some data indicating that trauma can sometimes cause the opposite effect, namely, if people suffering from trauma are able to comprehend and prevail over adversity, they can improve their mentalizing skills in a process called posttraumatic growth [6,34].

Research on mentalizing emphasizes difficulties associated with the tools used to measure this ability, including, firstly, the fact that some tools allow for only fragmentary measurements (e.g., “Reading the Mind in the Eyes” test—recognizing emotions, Strange Stories Test—understanding false beliefs, etc.), and secondly, that they refer to mentalizing in an overly simplified way, considering it only in terms of correct–incorrect [12]. Moreover, there exist many factors affecting replies provided by respondents. Finally, we lack tests to measure this ability in a way that reflects the complicated nature of social interactions and interpersonal functioning in real life [22]. One of the tools with high ecological validity is MASC (The Movie for the Assessment of Social Cognition), which makes it possible to assess both cognitive and affective components of mentalization by requiring subjects to attribute internal states to movie characters who interact with each other in situations strongly resembling real life [22,35]. MASC allows for a more detailed assessment of the mentalizing ability by classifying its impairments into three categories: hypermentalizing (overattribution of mental states to others), hypomentalizing or undermentalizing (insufficient description of mental states), and no mentalizing (total lack of mentalizing ability) [35,36].

When discussing the development of mentalizing abilities, the influence of factors such as age and gender is also worth mentioning. Research shows that the ability to mentalize positively correlates with age [22,37,38] and that it depends on gender, with women potentially being better at social cognition tasks. Nevertheless, the studies on gender correlation among adults do not provide clear results [22]. The above-mentioned interrelationships also apply to adolescents. A survey by Poznyak et al. among teenagers aged 12–17 shows that older adolescents make fewer hypomentalization mistakes. Moreover, with age, girls are statistically more correct and boys tend to commit more hypermentalization errors [22].

The capacity to understand each other’s goals, emotions, and desires enables not only complex collaboration and cooperation, but also a transmission of common goals, motives, values, and knowledge between generations through social learning [8,39]. Mentalizing makes establishing meaningful interpersonal relationships possible. Moreover, the related pursuit of cooperation and collaboration helps mitigate competitive and aggressive behavior [8]. People with a high mentalizing capacity show resilience in stressful situations. They are able to create a positive narrative of their lives despite the difficulties they experience [27]. They also exhibit the ability to explore and reflect both on the outside world and on their own psychic reality, which is represented in their visible creativity, their ability to symbolize and perceive life from different perspectives, their interest in dreams, fantasies, art, and music, and their curiosity about the inner world of other people [27]. On the other hand, mentalizing enables some people to take advantage of others through manipulation and deception [8].

## 3. Borderline Personality Disorder

Borderline personality disorder (BPD) is a severe mental disorder characterized by emotional dysregulation, identity disturbances, interpersonal relationship difficulties, abandonment fears, a chronic feeling of emptiness, suicidal and self-harm behaviors, inappropriate and intense anger, marked impulsivity, and transient, stress-related paranoid ideations as well as severe dissociative symptoms [1,3].

Formerly, it was assumed that patients must be above 18 years old to be diagnosed with borderline personality disorder. Currently, diagnosing BPD among adolescents is perceived as justified [3,40,41]. The Diagnostic and Statistical Manual of Mental Disorders 5th Edition allows for the diagnosis of BPD in this group if they present not less than five of the above-described symptoms for a period of at least one year [1,40,42].

The stability of the diagnosis among adolescents is similar to that of adults [3]. Evidence shows that BPD diagnosis in youth remains stable throughout a single year [43]. Other research, though, indicates that BPD symptoms significantly lessen from adolescence to adulthood while still maintaining rank-order stability [3,44].

When it comes to the prevalence, early studies show a higher percentage among community samples—from 11% to 26.7% of adolescents suffer from BPD [3,45,46], while more recent studies demonstrate a lower occurrence, between 0.06 and 3.27% [3,40,47]. Notably, the literature emphasizes the alarmingly high frequency of borderline personality disorder among teenagers hospitalized in psychiatric wards—studies demonstrated that up to 50% of these patients meet the criteria for BPD [3,41,48,49]. Research indicates that even as many as 78% of young people reporting to emergency rooms due to suicidal behaviors can exhibit symptoms of this disorder [40,50].

The issue of the clinical presentation of borderline personality disorder among adolescents is worth discussing, especially since diagnosing this age group can constitute a significant challenge [40]. Symptoms most indicative of BPD during adolescence include affective instability, a chronic feeling of emptiness, inappropriate, intense anger, identity disturbance (among girls), and paranoid ideations (among boys) [3,51,52]. Research shows that it is possible to distinguish the normative development of an adolescent from the one that indicates the development of BPD [42,53]. Potential difficulties associated with this differentiation used to constitute an argument against making a diagnosis among this population [3]. Characteristics specific to adolescents, such as impulsivity, a turbulent process of identity formation, and emotional instability, which may cause diagnostic difficulties, should decrease over time in healthy young people, while they persist or even worsen in those with BPD [42,54]. Moreover, teenagers with borderline personality disorder show more significant difficulties in interpersonal relationships than their healthy contemporaries in family and peer relations [55]. It has also been described that they experience more conflicted and violent romantic relationships and lower levels of intimacy [56,57,58,59].

Particular attention should be paid to the high prevalence of self-injury and suicide attempts among patients with BPD [2,53]. Goodman et al. showed that both in a group of adolescents and adults with borderline personality disorder, about 90% of patients report self-harm and over 75% admit that they have attempted suicide (76% in the case of teenagers versus 79% in the case of adults), most often through overdosing (over 50%). Moreover, in both groups, more than 88% of patients have a history of multiple self-mutilation behaviors, and above 50% made multiple suicide attempts [60]. Nevertheless, there are some differences in terms of self-aggression between these populations. The authors demonstrated that adolescents with BPD, significantly more often than adults, engage in frequent self-harm behaviors, while adults report more suicide attempts [60]. It is worth emphasizing that as much as 10% of people with BPD will die due to suicide [61,62].

Studies identified significantly higher co-occurrence of BPD in adolescent outpatients with both internalizing disorders (including mood and anxiety disorders) and externalizing ones (substance abuse and disruptive behavior disorders) in comparison to adolescents who have other personality disorders and the ones with proper personality development [63,64]. Similar results have been obtained among adolescent inpatients with BPD compared to those without this diagnosis [3,65]. The above-mentioned results reflect the outcomes of studies conducted on adults with BPD [66]. Furthermore, research shows that internalizing and externalizing disorders can often predict further development of BPD [64,67]. Presently, it is recognized that borderline personality disorder lies at the intersection of internalizing and externalizing disorders, not belonging to any of these groups, despite exhibiting characteristics of both and having high comorbidity with both of them [64].

Longitudinal studies on the course of BPD demonstrate that during the period from adolescence to adulthood, there is a change in the manifestation of the symptoms of the disorder—teenagers face mainly emotional dysregulation, impulsiveness, and suicidal behavior, whereas adults experience more prominent difficulties in interpersonal relationships and persistent functional impairment, going through alternating periods of remission and re-emergence of BPD symptoms [42,68,69]. The literature describes a gradual decrease in BPD symptoms during adulthood [42,70]. It includes a study by Gunderson et al. evaluating patients with BPD aged 18 to 45, which reported that 85% of them had a symptom remission over a ten-year period [68].

The etiology of borderline personality disorder has not yet been clearly explained [2]. However, it is considered to be complex and multifactorial [71]. BPD is seen as a developmental disorder whose symptoms begin to manifest clearly during adolescence [3,4]. Currently, it is generally accepted that BPD is conditioned by interactions of genetic, neurobiological, and environmental factors, including those related to the influence of family and peers [2,3,70]. The development of BPD should be seen from a transactional perspective. People are affected by their social environment, and at the same time, they influence that environment; thus, they are both recipients and senders of various messages and then respond to reactions that they themselves have provoked [72].

Research shows that BPD is moderately heritable among adults, with no specific genes yet identified [2,3,73]. Imaging studies in adolescents with BPD identified similar disturbances as in adults: a decrease in the volume of the frontolimbic network, including the orbitofrontal cortex and anterior cingulate cortex [3,74,75]. There is also some evidence demonstrating possible associations of improper levels of neuropeptides such as oxytocin and vasopressin with the development of BPD; however, there are not enough data to fully understand this connection [76]. Moreover, research emphasizes a potential role of the hypothalamic–pituitary–adrenal axis [3,40]. Blocked or delayed release of cortisol is seen in adults with BPD exposed to psychosocial stress. Impairments in the functioning of this axis have also been demonstrated in adolescents with borderline personality disorder. It is postulated that hypersecretion of cortisol may be related to the maladaptive stress responses in individuals with this disorder [3].

As mentioned before, studies indicate that social factors play an essential role in the etiology of BPD; however, they also underline the lack of specificity of these factors and their occurrence in other psychiatric disorders [67]. It has been shown that non-adaptive patterns of relationships between a child and its parents may contribute to the development of BPD [29,77]. People with BPD describe their relationships with their mothers as conflictual, distant, or overprotective and with their fathers as less engaging and more distant [5].

Research on the etiology of borderline personality disorder emphasizes a special role of attachment and its impairments; however, the specific character of these disturbances is not yet fully established. According to attachment theory, insecure attachment styles are caused by the child’s attempts to maintain a sense of security in a situation of incorrect parenting [78]. It has been hypothesized that improper relationships with early attachment figures (primary caregivers) may contribute to disturbed mental representations. The probable connection between disorganized attachments in childhood and borderline personality disorder in adulthood is indicated by research demonstrating that self-representations play the role of a mediator between the above-mentioned variables [79,80]. However, it is also suggested that a secure attachment to one parent may play a potential protective role in the presence of a disorganized attachment to the other parent. This hypothesis has been confirmed by research showing that adolescents with BPD are characterized rather by the existence of a disorganized attachment not with one but with both parents [81]. Moreover, there are some data indicating that preoccupied attachment during early adolescence precedes improper behaviors exhibited by teenagers, like sexual risk taking and aggression, which are similar to some symptoms of BPD. For this reason, a possible relationship between preoccupied attachment and borderline personality disorder has been postulated [80,82]. Some available studies confirm the occurrence of preoccupied attachment in adults with borderline personality disorder and in adolescents with this diagnosis [81,83].

The possible involvement of aversive childhood events in the development of BPD has also been identified [3,29]. Porter et al., in their meta-analysis, demonstrate that considering case–control research, individuals with BPD are 13.91 times more likely than non-clinical controls and 3.15 times more likely than other psychiatric groups to report childhood adversity (including physical, emotional, and sexual abuse, as well as physical and emotional neglect) [84]. However, it has been shown that borderline personality disorder is correlated with increased rates of all included subtypes of adversity, with the most visible effects in the cases of emotional abuse and neglect [84]. The relationship between adverse experiences in childhood and BPD symptoms is also noticeable when analyzing a group of young people under 19 years of age [85]. The meta-analysis based on studies in this age group indicates that among adolescents (13–19 years old), sexual violence increases the likelihood of a diagnosis of BPD by more than 5 times, while physical violence increases it by 2.6 times. Moreover, similar associations have been demonstrated for teenagers experiencing maternal hostility/verbal abuse and neglect. Each form of the aforementioned maltreatment increases the risk of being diagnosed with BPD almost fivefold [85]. One of the theories explaining the difficulties in interpersonal relationships manifested by people with BPD suggests that they result from the necessity for children who experience violence to adjust to their living conditions by becoming more sensitive and reactive to threats [5]. In this context, their increased vigilance can be seen as a form of social adaptation to the hostile environment. In a situation of an adult having malevolent feelings and intentions towards a child, in order to survive, the child needs to cease understanding mental states of the aggressive adult [3]. In addition, research demonstrates that childhood trauma affects neurotransmission, including glutamatergic, serotonergic, dopaminergic, and adrenergic transmitters, which may indicate that BPD symptoms result from these dysfunctions [86]. However, currently, it is recognized that experiencing violence by a child is neither a necessary nor a sufficient condition for the development of BPD and that attention should be paid to many aspects of the relationship between the child and parents, including the factors that may predispose this relationship to the emergence of violence and how caretakers respond to its occurrence [5].

Notably, the possible role of experiencing bullying and rejection by peers in developing borderline personality disorder is now also emphasized [87].

Currently, research highlights similarities between traits of borderline personality disorder and complex posttraumatic stress disorder (cPTSD) [88,89]. According to the ICD-11 classification, the criteria for complex PTSD include, on the one hand, the same criteria as for PTSD, and, on the other hand, symptoms of three domains of Disturbances in Self-Organization (DSO), like emotional dysregulation, negative self-perception, and persistent problems in interpersonal relationships [88]. It is argued that DSO significantly overlaps with features of BPD [89]. However, existing evidence suggests, firstly, that symptoms of DSO are distinguishable from those of borderline personality disorder and, secondly, that it is less common for borderline features to be manifested alone and not in combination with symptoms of PTSD and/or cPTSD. What is worth mentioning is the co-occurrence of BPD symptoms with PTSD and/or cPTSD manifestation, which indicates the experience of more severe cumulative childhood interpersonal trauma than in the case of PTSD and/or cPTSD alone [88,90].

## 4. Mentalizing and Borderline Personality Disorder

Many studies confirmed that adults with borderline personality disorder experience impairments in the ability to mentalize, which led to the creation of a mentalizing model of borderline personality disorder, according to which mentalization disturbances in situations of attachment-related stress or arousal are the core feature of BPD [5,9,27].

Fonagy and colleagues hypothesize that there are several paths of BPD development, conditioned by interactions between environmental and biological factors, which include people at increased risk of developing BPD due to mentalization disorders (resulting from being brought up in families characterized by low levels of mentalization and lack of attention paid to internal states), but also people who show inhibition of mentalization due to experiencing violence and rejection [91]. Moreover, these authors assumed that borderline personality disorder is associated with attachment disorders and that patients with BPD suffer from hyperactivity of the attachment system, expressed in desperate attempts to avoid abandonment, in unstable and intense interpersonal relationships, and in a very rapid transition to intimacy in relationships. According to their hypotheses, hyperreactivity of the attachment system is associated with traumatic experiences, which by contributing to impairments in the ability to mentalize, may be one of the factors leading to the development of BPD [5,91]. Studies confirm the occurrence of insecure attachment styles in adults with borderline personality disorder [28,59] and indicate that attachment disturbances in children and adolescents may be associated with a later diagnosis of BPD in adulthood [59,79].

Fonagy et al. suggest that in the case of borderline personality disorder, there is a low threshold for the deactivation of mentalizing in a controlled manner, especially in terms of distinguishing one’s own mental states from those of other people. It results in experiencing interpersonal situations and internal states in a way that is unbearable for people with BPD, which, in turn, causes disturbances in other dimensions of mentalizing [91]. Loss of mentalizing capacity leads to pre-mentalizing modes of functioning, such as psychic equivalence (concreteness of thought, a state in which thoughts and feelings lose their “as if” quality and everything seem to be “real”), pretend mode (state of dissociation in which a person experiences the inner world as having no connection with the external world and in which thoughts and feelings seem to be almost meaningless), and teleological mode (actions are outcome-oriented, where visible physical reality becomes the only criterion of truth) [5]. Pre-mentalizing modes of functioning result in susceptibility to engaging in destructive interpersonal cycles characterized by significant affective dysregulation [91]. Research indicates that mentalization dysfunctions contribute to BPD patients’ interpersonal relationship problems [74,92], including the strong fear of rejection that they experience [92,93,94]. To cope with interpersonal dysfunction and distress, impulsivity, affect dysregulation, and the feeling of inner emptiness, patients with BPD rely on maladaptive emotion regulation strategies, such as self-harm, substance abuse, and hypersexuality [91].

Studies indicate the potential mediating effect of Early Life Stress (ELS) on the relationship between borderline personality disorder and impairments in the ability to mentalize [95]. It is postulated that ELS, comprehended as stressful and adverse experiences in the period from fetal life to childhood, contributes to the development of numerous mental disorders, including borderline personality disorder [96,97]. Research demonstrates that ELS predominantly negatively influences cognitive brain networks (including those based on the hippocampus and prefrontal cortex) and a network involved in social behaviors [98,99,100].

Despite many studies confirming the occurrence of mentalization impairments among people with BPD [36,101,102,103,104], data demonstrating the lack of this association are also available. Some research shows that adults with borderline personality disorder are characterized by a greater aptitude for recognizing mental states based on pictures of the eye area in the “Reading the Mind in the Eyes” test (RMET) than healthy controls [105,106] and that they exhibit an increased ability to mentalize measured by other tests [107]. There is also evidence indicating that people with BPD do not differ significantly from healthy people regarding this ability [108,109,110].

Several reasons for the aforementioned differences can be listed: problems with recognizing different levels of complexity of mentalizing capacity, arousal induced during measurement, and ecological validity of the assessments used to evaluate mentalizing ability [12,59,104]. It has been implied that studies which do not demonstrate mentalizing ability disorders among people with BPD are not able to do so, as they use measurement tools that lack complexity and ecological validity and because they are not able to evoke increased levels of arousal in subjects [36]. Jańczak points to three possible causes for the divergent results of studies on mentalization in people with BPD. Firstly, these studies measure different aspects of mentalization, only some of which are disturbed in these people. Secondly, mentalization should be treated not only as a feature but also as a state that depends on measurement conditions. The last possible cause is the influence of other factors on the capacity to mentalize [111]. Moreover, an enhanced ability of people with BPD to understand social cues, demonstrated in some studies, can be considered from the perspective of Marsha Linehan’s biosocial theory, according to which these individuals show increased sensitivity to emotional stimuli, especially social rejection and threat [59,112].

It is also worth emphasizing that differences between the results of MASC and other tests assessing mentalizing in people with BPD may be related to the fact that mentalization disorders in this group are only revealed when people suffering from BPD must engage higher-order cognitive abilities to integrate many different social cues (related to the emotional expression of the face, body language, gestures, tone of voice, literal statements, and metaphors), which forces them to use automatic mentalization [12]. Fonagy et al. suggested that people with borderline personality disorder have difficulty adjusting a type of mentalization along a controlled–automatic dimension to a particular social situation, which becomes especially evident when they experience high levels of arousal [113]. In cases where rapid, automatic mentalization is required, for example, during contact with attachment figures, individuals with BPD may overuse controlled processing, while in situations where controlled mentalization would be adequate, they excessively rely on automatic processing [59,113].

Although many studies confirm mentalizing dysfunctions among adult patients with BPD, they also give ambiguous results concerning the type of impairment. Some studies primarily indicate hypomentalizing manifested by these patients [92,114], while other research, based mainly on the previously mentioned MASC, shows mostly hypermentalization errors [36].

Sharp and colleagues created a hypermentalizing model which indicates that hypermentalizing is the main type of mentalizing impairment among people with BPD [59]. Hypermentalizing is defined as overattribution or excessive inference about other people’s mental states that is not based on observable evidence available to others [35,36,59]. The aforementioned model implies that people with BPD have difficulty flexibly employing the four dimensions of mentalizing, and instead, they overuse one of the extremes of these dimensions. Problems they experience in this regard become evident in situations of emotional arousal or attachment hyperactivation. In such cases, people with BPD show limited ability to self-monitor or use flexible mentalization. Impairments in employing the self vs. others mentalizing lead to projecting one’s own mental states onto other people, manifesting in hypermentalization [36,59].

Initially, studies seemed to provide evidence to support this theory [115,116]. The meta-analysis presented by McLaren et al. confirms the occurrence of hypermentalization measured in MASC studies in both adults and adolescents with this diagnosis [36]. On the other hand, it presents evidence that hypermentalization is not specific to BPD but rather characteristic of general psychopathology. The authors point out that it is currently impossible to clearly state whether this is due to different mechanisms in different disorders or whether these results should be treated as a manifestation of one cross-diagnostic mechanism. Moreover, they also suggested a possibility that hypermentalization is specific to borderline personality disorder, assuming that the features of BPD are also visible in other mental disorders, which would be the reason for this type of mentalization impairment also in patients with other diagnoses [36]. This hypothesis is supported by the results of the research showing that borderline personality disorder is strongly related to the p-factor (a common factor of psychopathology, which potentially indicates a risk of developing any form of a mental illness) [36,117] and the general factor of personality pathology (which represents criterion A from the alternative model of personality disorders) [36,118].

Research suggests that mentalization disorders in the form of hypermentalization may be both a consequence and one of the causes of affect dysregulation observed in patients with BPD. In the available literature, on the one hand, it is recognized that strong emotions may lead to “switching off” the ability to mentalize cognitively and may contribute to disorders in this capability in the form of hypermentalization. On the other hand, it is emphasized that strong, negative affective states in patients with BPD may be caused by a distorted understanding of other people’s behaviors and internal states, e.g., by perceiving non-existing hostility or rejection [80,119]. Moreover, it is postulated that the negative affective states induced in this way trigger impulsive, aggressive, and self-aggressive behaviors exhibited by people with BPD, which, additionally, are likely to generate difficulties in interpersonal relationships [78]. It seems to be justified to consider the above-mentioned phenomena as a kind of vicious circle in which mentalization disorders cause emotional dysregulation and together they both induce problems in relationships which, in turn, strengthen negative affective states, leading to further deterioration in the ability to mentalize.

There are just a few studies on the issue of mentalization disorders in adolescents with borderline personality disorder. The meta-analysis mentioned above takes into account six studies on this population (under 18 years of age) diagnosed with BPD [47,120,121,122,123,124], and it also refers to two population studies on adolescents with other mental disorders (major depressive disorder, generalized anxiety disorder, attention-deficit hyperactivity disorder, somatic disorders, schizophrenia, conduct disorder, autism spectrum disorder) [36].

A study by Von Ceumern-Lindenstjerna et al. demonstrated that teenage girls with BPD exhibit greater vigilance to negative emotional facial expressions than healthy adolescents, but they do not differ in this respect from teenagers with other mental disorders [125].

Sharp et al., in the first research using MASC in a group of adolescents with borderline personality disorder (62 girls and 49 boys), demonstrated that hypermentalizing shows a strong correlation with BPD features, while there is no such association for hypomentalizing and no mentalizing [115]. Moreover, results indicated that the connection between hypermentalizing and borderline features was partially mediated by emotional dysregulation [115]. In a study described by Sharp et al. two years later, conducted among adolescents admitted to a treatment program of a private tertiary care inpatient treatment facility, it has been shown that there are significant differences between adolescents with BPD and those without this condition for hypermentalizing at admission, but not at discharge [116].

Sharp et al., in a study from 2016 on a group of 259 adolescents aged 12 to 17, demonstrated that increased severity of borderline features was significantly correlated with hypermentalizing, emotional dysregulation, internalizing, and externalizing symptoms. In addition, it was also found that greater attachment coherence has a negative association with hypermentalizing [124]. What is more, the research also confirmed that hypermentalizing and emotion dysregulation jointly mediate the relationship between attachment coherence and borderline features [124].

Quek et al. found that there is no statistically significant difference between the results of adolescents with BPD and healthy adolescents from the control group in the RMET test, while this difference was demonstrated in MASC and the Reflective Function Questionnaire for Youth (RFQ-Y) [123]. The MASC revealed that adolescents with BPD exhibit mentalization dysfunction in the form of hypermentalization, which significantly distinguishes them from a group of healthy teenagers. It was also discovered that adolescents with BPD show a significantly lower capacity to mentalize in the context of attachment relationships, operationalized as reflective function (RF), compared to healthy adolescents (based on the RFQ-Y). RFQ-Y assesses the ability to mentalize oneself and others, while RMET and MASC refer to mentalizing other people’s mental states. For this reason, this study shows that adolescents with BPD also have difficulty mentalizing with regard to themselves. At the same time, it has been emphasized that the assessment of this dimension of mentalization is more difficult and potentially burdened with a more significant error due to the use of a self-report tool [123].

Duval et al., in a study of 150 adolescents and young adults aged 12 to 21, showed that borderline personality features correlate with hypermentalizing measured by MASC and with more uncertainty/confusion and lower interest/curiosity measured by RFQ-Y [120].

Goueli et al. demonstrated in their research that teenage girls meeting the criteria of BPD exhibit disorders in understanding social situations (an impaired ability to recognize mental states based on pictures of the eye area in RMET and hypermentalizing measured by MASC) and that severity of disturbances in mentalizing increased with the severity of borderline symptoms [121]. A negative correlation was found between impulsiveness, a sense of emptiness, and quasi-psychotic symptoms. The authors postulate that the severity of these symptoms may also negatively impact mentalization abilities (which is essential from the perspective of transactional understanding of the impact that BPD and mentalization disorders have on each other) [121].

Soomma et al. conducted a cross-cultural replication of Sharp and colleagues’ (2011) findings in a group of 58 Italian adolescents with BPD with a comparison to healthy controls, which confirmed that there is a significant association between borderline traits and hypermentalizing [47].

Norup and Bo, in a study involving 109 adolescents aged 13–18, using RFQ-Y, demonstrated that mentalization acts as a mediator between the features of borderline personality and externalizing and internalizing disorders [64]. It was also found that greater severity of BPD features is associated with greater severity of psychopathology and lower mentalization abilities [64].

Available research indicates that cultural factors may influence both symptoms of borderline personality disorder and the ability to mentalize [47,126]. A systematic review of research on mentalization in the context of cultural factors by Aival-Naveh E, Rothschild-Yakar L, Kurman J demonstrates that people from collectivistic cultures exhibit a different course of development of the capacity to mentalize than people from individual-oriented communities [126]. It is indicated that the above-mentioned differences may result from various ways of bonding with children in these cultures. Some authors postulate that in individualistic societies, in order to build an appropriate perception of others as a credible source of knowledge, a child needs to be involved in a dyadic interaction with its primary caregiver, within which the child’s internal states are properly reflected by its caregiver. On the other hand, in collectivistic societies which teach children to perceive themselves as a part of a broader social system, it seems to be evolutionarily beneficial for a child to quickly learn to pay attention to mental states of other people, which, in turn, generates the ability to trust others as a reliable source of knowledge. Consequently, in collectivistic cultures, more attention is attracted to other persons’ internal states, which may contribute to an enhanced capacity to mentalize with others, while people from individualistic cultures tend to focus more on their own internal states, which results in a better ability to mentalize with themselves [126,127]. However, it has been highlighted that these observations require further research [126,128]. It is hypothesized that the aforementioned cultural differences may lead to various patterns of mentalization dysfunctions among adolescents with borderline personality disorder in different cultures. The need for further research to comprehensively understand the cross-cultural aspects of mentalization in adolescents with BPD should be emphasized. Such studies may contribute to devising a culturally sensitive treatment that could be significantly more efficient.

The results of studies on mentalizing disorders in adolescents with borderline personality disorder seem to mirror what has been demonstrated by research carried out among the population of adults with BPD [36]. However, when interpreting studies regarding teenagers, it seems important to take into account specific developmental aspects of adolescence. It is worth emphasizing that adolescence is a period of intense emotional and social development, which means that some symptoms characteristic of BPD may just be manifestations of normal developmental processes, which, consequently, implies the necessity to consider age-appropriate behaviors while diagnosing teenagers [21,22,42]. The available literature, based on longitudinal research, shows that symptoms of BPD vary at different stages of development [42,68,69]. However, specific features of borderline personality disorder among adolescents may continue to be manifested in adulthood, especially if young patients do not undergo appropriate treatment which, when provided, may contribute to the reduction in the severity of symptoms in the course of the disorder [129,130]. In order to explore the full scope of mentalization disorders among people with BPD and to show the development of their mentalizing ability, further research, including longitudinal studies, seems to be necessary.

## 5. Clinical Implications

Understanding mentalizing disorders in people with BPD, including the profile of these disorders in adolescents, may contribute to the creation of tests that will enable the detection of BPD in the early stages of development, perhaps even before the full manifestation of symptoms. Consequently, by early implementation of appropriate therapeutic techniques, it may be possible to achieve remission of symptoms or to prevent their full manifestation. Furthermore, early therapeutic interventions may reduce the risk of self-aggressive behaviors, including suicide attempts, as well as significantly improve the functioning of people with BPD, by helping them avoid long-term negative effects of the disorder, such as persistent difficulties in social relationships and disturbed school and professional functioning.

Based on the model linking mentalization disorders and symptoms of borderline personality disorder, Anthony Bateman and Peter Fonagy created mentalization-based therapy [6,130]. Although the available evidence on its effectiveness among adults and adolescents is promising, the need for further research is emphasized to fully determine the efficacy of this form of therapy in people with BPD, including adolescents with this disorder [130,131].

## 6. Conclusions

Research indicates that adolescents with borderline personality disorder exhibit impairments in the ability to mentalize in the form of hypermentalizing, defined as an overattribution of mental states to other people. Based on the available literature, it cannot be concluded that a tendency to hypermentalizing is a specific feature of teenagers with BPD. Studies demonstrate that this kind of mentalizing impairment also occurs in people with other mental disorders. Moreover, there is evidence that adolescents with BPD have a reduced capacity to mentalize their own inner states. More research is needed to fully assess the profile of the mentalizing abilities of adolescents with this condition.

## Data Availability

Not applicable.
